# A Single-Nucleotide Polymorphism of Human Neuropeptide S Gene Originated from Europe Shows Decreased Bioactivity

**DOI:** 10.1371/journal.pone.0083009

**Published:** 2013-12-27

**Authors:** Cheng Deng, Ximiao He, Aaron J. W. Hsueh

**Affiliations:** 1 Program of Reproductive and Stem Cell Biology, Department of Ob/Gyn, Stanford University School of Medicine, Stanford, California, United States of America; 2 National Cancer Institute, National Institutes of Health, Bethesda, Maryland, United States of America; Institute of Hydrobiology, Chinese Academy of Sciences, China

## Abstract

Using accumulating SNP (Single-Nucleotide Polymorphism) data, we performed a genome-wide search for polypeptide hormone ligands showing changes in the mature regions to elucidate genotype/phenotype diversity among various human populations. Neuropeptide S (NPS), a brain peptide hormone highly conserved in vertebrates, has diverse physiological effects on anxiety, fear, hyperactivity, food intake, and sleeping time through its cognate receptor-NPSR. Here, we report a SNP *rs4751440* (L^6^-NPS) causing non-synonymous substitution on the 6^th^ position (V to L) of the NPS mature peptide region. L^6^-NPS has a higher allele frequency in Europeans than other populations and probably originated from European ancestors ∼25,000 yrs ago based on haplotype analysis and Approximate Bayesian Computation. Functional analyses indicate that L^6^-NPS exhibits a significant lower bioactivity than the wild type NPS, with ∼20-fold higher EC_50_ values in the stimulation of NPSR. Additional evolutionary and mutagenesis studies further demonstrate the importance of the valine residue in the 6^th^ position for NPS functions. Given the known physiological roles of NPS receptor in inflammatory bowel diseases, asthma pathogenesis, macrophage immune responses, and brain functions, our study provides the basis to elucidate NPS evolution and signaling diversity among human populations.

## Introduction

In two randomly selected human genomes, 99.9% of the DNA sequence is identical and the remaining 0.1% accounts for variations among individuals [1]–[3]. SNP (Single-Nucleotide Polymorphism) is the simplest form of genetic variations, occurring at a frequency of about 1 per 1,000 bp in human chromosomes [4]. Identifying important SNPs which underlie regional adaptations is a key to understanding genetic diversities among different human populations. More and more adaptive traits resulting from one single SNP have been elucidated in selective human populations. For polypeptide ligands and receptors, a GIP (gastric inhibitory polypeptide) SNP with altered bioactivity was shown to be the result of adaptive selection in Eurasian populations [5] whereas an SNP of the EDAR gene was found to increase the number of active eccrine glands in the Han Chinese [6]. In addition, a Leu7Pro polymorphism in the signal peptide region of the NPY (neuropeptide Y) gene has been found to show higher plasma NPY levels in response to physiological stress and is associated with a greater risk of developing alcohol dependence in patients [7]. Also, two SNPs found in the chemokine receptor CX3CR1 gene of Caucasians were shown to be more responsive to the chemokine ligand fractalkine and confer rapid progression to AIDS [8]. With the completion of more SNP projects, we can better elucidate the genetic diversity of polypeptide ligands among different human populations during evolution.

Neuropeptide S (NPS) is a conserved 20-amino-acid peptide found in mammals and identified as a neuromodulator expressed in the brainstem [9]. *In vitro* studies demonstrated that NPS binds specifically to NPSR, a G protein-coupled receptor, to increase cAMP production and intracellular Ca^2+^ levels [9], [10]. NPS and its receptor-NPSR are expressed in various tissues in rodents, with the highest levels in brain, thyroid, salivary, and mammary glands [9]. In the brainstem, the NPS highly expressed in three groups of neurons located between the locus coeruleus (LC) and Barrington's nucleus. The first and second groups of NPS-expressing neurons co-expressed with the glutamatergic neurons and are localized to the locus coeruleus and principle sensory nucleus, respectively. In contrast, the third NPS-expressing neuron group is localized to the lateral parabrachial nucleus and later is indentified to co-express the corticotropin-releasing factor (CRF) [9], [11]–[14]. In contrast to NPS, its receptor NPSR shows a wider expression pattern in the brain. The highest expression of NPSR was found in areas involved in olfactory function, including the anterior olfactory nucleus, the endopiriform nucleus, and the piriform cortex [11]–[13]. NPSR is also expressed in several brain regions mediating anxiety responses, including the amygdaloid complex and the paraventricular hypothalamic nucleus [11]–[13]. Also, NPSR is expressed in regions involved in sleep neurocircuitries, such as the thalamus, the hypothalamus, and the preoptic region, as well as in the output and input regions of hippocampus, including the parahippocampal regions, the lateral entorhinal cortex, and the retrosplenial agranular cortex [11]–[13].

The wide expression pattern of NPSR implies that the NPS/NPSR system may be important for diverse brain functions. *In vivo* studies in mice showed that treatment with NPS suppresses anxiety and appetite [9], [15], [16], induces wakefulness and hyperactivity [17], decreases conditioned fear responses [12], stimulates the hypothalamic-pituitary-adrenal axis [18], and inhibits food intake [18].

Here, we found that a SNP *rs4751440* causing non-synonymous substitution at the 6^th^ position (V to L) of the NPS mature peptide region has a relatively higher allele frequency in Europeans. This SNP variant (L^6^-NPS) shows a decreased ability to activate the NPSR receptor.

## Materials and Methods

### Haplotype Analysis

SNPs data from 1,092 samples were downloaded from the 1,000 Genome Project. After excluding monomorphic SNPs and SNPs with inconsistent genotypes, we obtained a final data set of 888 SNPs in 1,092 samples (2,184 chromosomes) from 14 populations. We inferred haplotype data by phasing with fastPHASE [19]. Examination of linkage disequilibrium patterns in the flanking regions revealed a ∼14 kb block surrounding *rs4751440*. We counted the number of chromosomes for each haplotype in individual populations and plotted the haplotype frequencies on a world map.

### Approximate Bayesian Computation

We used the spatially explicit population model [20] by considering evolutional process such as population structure, drift, and natural selection, as well as various demographic processes including population growth, sporadic long-range migration, and the effects of the spread of farming on carrying capacities, to implement the forward simulation. We applied an Approximate Bayesian Computation (ABC) inference framework to estimate parameters of interest [6], [21]. Briefly, we employed spatially explicit forward simulations to model the origin and the subsequent spread of the derived *rs4751440* in Europe. The demic grid of this simulation model encompasses the geographic region between 35°N to 70°N, and 20°W to 60°E, covering all the available sampled populations in Europe. This geographic region was modeled as a series of 2,800 rectangular demes with each being one degree in latitude and one degree in longitude, which includes 1,573 land demes and 1,227 sea demes. The maximum population size in each deme (*K_deme_*) is calculated as described by Itan et al. (2009) [22]: *K_deme_* = (0.2*cl*+0.8*el*)*D_max_*A_deme_, where the *cl* is relative climiate, with values of 0.25, 0.5, 0.75 and 1 for polar, cold, dry, and temperate climates respectively; the *el* is relative elevation; the *A_deme_* is the area of the deme (km^2^), and the *D_max_* is the maximum population density which fixed as 5 individuals per km^2^. Our simulations start from 40,000 yrs BP (1,600 generations ago, assuming 25 yrs per generation), because modern humans originated ∼195,000 yrs ago in Sub-Saharan Africa and migrated towards Northern Eurasia ∼40,000 yrs ago [23], [24]. We assume the appearance of farmer as early as 7,000 yrs BP, and the allele of *rs4751440* appeared no later than 5,000 yrs BP. The population movements within and between demes, population growth and selection coefficient were simulated as described by Kamberov et al. (2013) [6]. At the end of each simulation, we recorded the parmeter values that generated the simulation, including the genereation and the location (which deme) where the allele originated, and the derived allele frequency in 5 locations where observed allele frequency data available. We compared summary statistics (allele frequency of *rs4751440* in 5 populations) recorded after each simulation to observed frequencies, and accepted only those simulations with sufficiently small differences. We calculated the Euclidean distance (δ) between the simulated and observed statistics for each simulated data set and maintained those simulations with the smallest values. Among the total 3,000,000 simulations performed, we present parameter estimates using the best 0.03% (top 1,000) of the simulations, as well as we chose to base our inferences on the best 0.17% (top 5,000) in order to avoid over-fitting.

### Materials

Full length cDNA for the NPSR receptor was purchased from OriGene Technologies. Different peptide variants (NPS, L^6^-NPS, A^6^-NPS, I^6^-NPS, F^6^-NPS, N^6^-NPS, K^6^-NPS, D^6^-NPS) were chemically synthesized by the Pan Facility at the Stanford University. In addition, wild type NPS and L^6^-NPS were chemically synthesized by NEO Group Inc. CRE- and NFAT luciferase reporter plasmids as well as the pSV-β-galactosidase control vectors were purchased from Promega.

### Luciferase Assays

HEK293T cells seeded in 24-well plates were co-transfected with different luciferase reporters (50 ng), the pSV-β-Gal plasmid (5 ng), and the NPSR receptor plasmid (50 ng). After 36 h, cells were cultured in serum-free media for another 18 h with increasing doses of the various NPS peptides. Luciferase activities were determined using the luciferase assay kit (Promega) and normalized using β-galactosidase activities. All experiments were performed at least three times in triplicates. Data (EC_50_) were analyzed using Graphpad Prism 5.0.

## Results

Based on the new SNP database of the NHLBI GO Exome Sequencing Project (ESP), we screened the human genome for SNPs in the mature regions of all polypeptide ligands deposited in the Human Plasma Membrane receptome Database (HPMR; http://receptome.stanford.edu/HPMR/) [25], [26]. As shown in Table S1, 339 SNPs and 59 SNPs were found to cause non-synonymous substitution in the mature regions in type A and B polypeptide ligands, respectively. Among different SNPs, we focused on the genotypic and allelic frequencies of the SNP *rs4751440*, locating on chromosome 10:129350856. This SNP was identified in both NHLBI GO Exome Sequencing Project (ESP) and International HapMap Project (Table 1 and 2). This SNP (G to C) causes non-synonymous amino acid substitution (V to L) in the NPS coding region. Both databases showed higher allele frequencies (about 13%) for L^6^-NPS in Europeans than other populations, with about 22% heterozygotes (NPS/L^6^-NPS) and about 2% homozygotes (L^6^-NPS/L^6^-NPS) in Europeans (Table 1 and 2).

**Table 1:** *Rs4751440* (L^6^-NPS) exhibits a high frequency in Europeans based on Exome Sequencing Project.

Exome Sequencing Project	Rs4751440 allele frequency		Rs4751440 genotypes		
Population	L^6^-NPS	NPS	C/C	G/C	G/G
EuropeanAmerican	13.95%	86.05%	1.8%	24.3%	73.9%
AfricanAmerican	2.2%	97.8%	0.1%	4.19%	95.7%

The SNP *rs4751440* allele frequency and genotypes were calculated based on Exome Sequencing Project.

**Table 2:** *Rs4751440* (L^6^-NPS) exhibits a high frequency in Europeans based on Exome Sequencing Project based on Hapmap project.

HapMap Project	Rs4751440 allele frequency		Rs4751440 genotypes		
Population	L^6^-NPS	NPS	C/C	G/C	G/G
EUR	12.7%	87.3%	2.11%	21.11%	76.78%
AMR	5.5%	94.5%	0%	11.05%	88.95%
AFR	0.4%	99.6%	0%	0.81%	99.19%
ASN	0%	100%	0%	0%	100%

The SNP *rs4751440* allele frequency and genotypes were calculated based on Hapmap project. Different human populations (AFR-African, AMR-Ad Mixed American, ASN-East Asian, EUR-European, SAN-South Asian) from the Hapmap project are shown.

**Figure 1:**
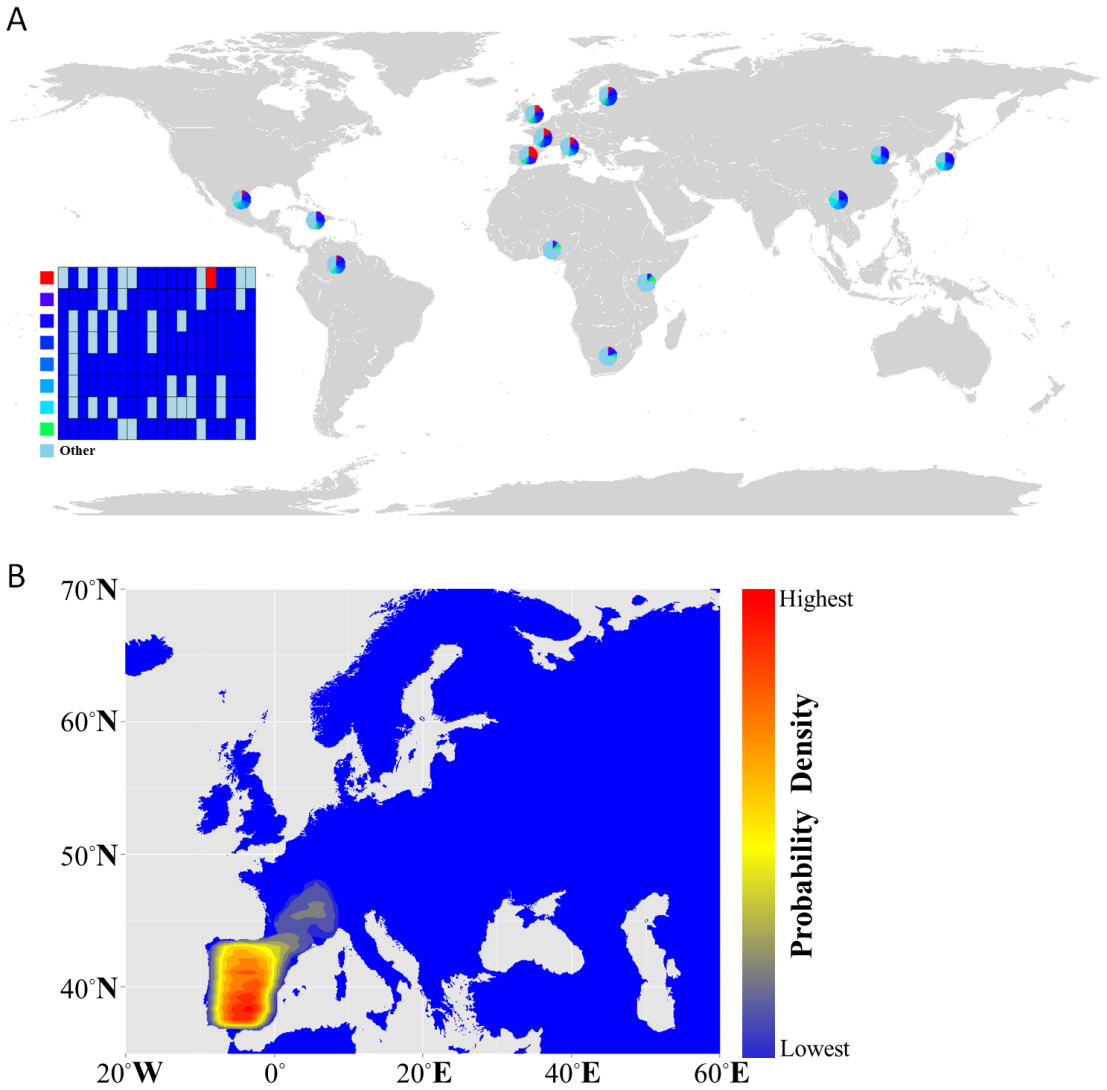
Origination of the L^6^-NPS variant. (**A**) Haplotype distribution of the genomic region surrounding SNP *rs4751440* based on 20 SNPs covering about 14 kb. The eight most common haplotypes are shown in the left inset, and the remaining low-frequency haplotypes are grouped as “Other.” The chimpanzee allele was assumed to be the ancestral one. The *rs4751440* is shown in red whereas all other alleles are in dark blue. (**B**) The approximate posterior probability density for the geographic origin of L^6^-NPS obtained by the ABC simulation. The heat map was generated using 2D kernel density estimation of the latitude and longitude coordinates from the top ranked 5,000 of 3,000,000 simulations. Red color represents the highest probability, and blue the lowest.

Using the publicly available data (HapMap Project and The 1,000 Genomes Project), we further examined a 500-kb genomic region that covers 888 SNPs flanking the L^6^-NPS SNP-*rs4751440* in 14 worldwide populations in order to investigate the origination of this SNP. Haplotype analysis suggested a single origin of the newly derived allele (Fig. 1A), with the mutation (G>C) locating in a unique, and nearly unbroken haplotype spanning ∼14 kb in the CEU (Utah Residents with Northern and Western European ancestry) population (Fig. S1). Under the neutrality rule, the average age of a polymorphism with the frequency *p* is estimated to be −4N*_e_*[*p*(log*p*)/(1-*p*)] [27], [28]. With the assumption of N*_e_* = 5,000 for each population, the time for the derived allele of *rs4751440* to arise to its current frequency in the AFR, AMR and EUR populations is ∼11,000, ∼43,000, and ∼160,000 yrs, respectively. However, these estimations are incompatible with archaeological evidence showing that modern humans originated ∼195,000 yrs ago in Sub-Saharan Africa and migrated towards Northern Eurasia ∼40,000 yrs ago [23], [24]. Alternatively, we estimated the age of *rs4751440*-assocatated haplotypes based on the decay of haplotypes. Assuming a recombination rate derived from estimates of linkage disequilibrium, *rs4751440*-assocatated haplotypes arose ∼27,000 yrs in the EUR population. We also performed another linkage disequilibrium analysis using a recombination rate ranging from 0.5–3.03 cM/Mb as used in previous studies [29]–[31] and an origin age of between 39,000 and 7,000 yrs was derived.

To better estimate the temporal and geographic origin for the *rs4751440* allele, we performed three million forward simulations using a spatially explicit population model [20] to identify the origination and spread of allele *rs4751440* in Europe. The Approximate Bayesian Computation (ABC) model [32] was used to compare simulated to observed allele frequencies and to estimate the evolutionary and demographic parameters [6]. The ABC modeling estimated that *rs4751440* originated in Western Europe between 12,250 and 39,000 yrs ago (95% credible interval), with a mode of 23,650 yrs ago and a median of 25,200 yrs ago (Fig. 1B, Fig. S2, Table S2). Combined with linkage disequilibrium analysis, the *rs4751440* probably originated from the ancestor of European population ∼25,000 yrs ago.

**Figure 2:**
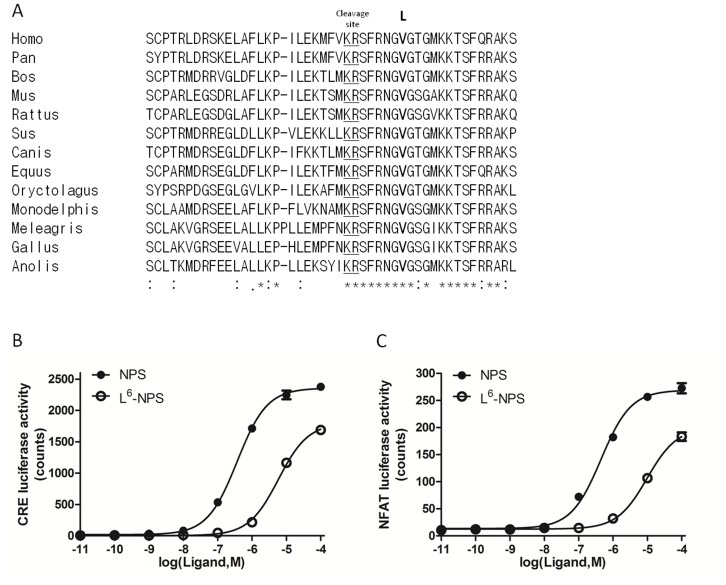
Sequence comparison and lower NPSR receptor signaling ability of the L^6^-NPS variant. (**A**) Alignment of NPS among diverse vertebrate species. Sequences were aligned by the ClustalW program [75]. Stars indicate identical amino acids and double dots (:) indicate conserved amino acids. The predicted convertase cleavage sites (KR) were underlined and the 6^th^ amino acid “V” or “L” in the mature peptide region are shown in bold. (**B**) Comparison of NPS and L^6^-NPS signaling based on the CRE-luciferase assay. (**C**) Comparison of NPS and L^6^-NPS signaling based on the NFAT luciferase assay. HEK293T cells seeded in 24-well plates were co-transfected with different luciferase reporters (50 ng), the pSV-β-Gal plasmid (5 ng), and the NPSR receptor plasmid (50 ng). After 36 h, cells were cultured in serum-free media for another 18 h with increasing doses of the various NPS peptides. EC_50_ values were analyzed using Graphpad Prism 5.0.

Non-synonymous mutations on mature region of peptide ligands could alter their bioactivities. The proprotein NPS is cleaved to the functional mature peptide with 20 residues [9] (Fig. 2A). The matured NPS shows high conservation in vertebrates and the *rs4751440* SNP generates a non-synonymous substitution (V to L) on the 6^th^ residue of NPS. This position is identical in all vertebrate species examined (Fig. 2A). The 6^th^ valine residue is located in the hinge region of NPS, thus important in maintaining its proper conformation and mutagenesis experiments indicated this position to be essential for interactions with the NPSR receptor [33], [34].

To compare the bioactivity of wide type NPS (NPS) and the SNP variant (L^6^-NPS), we monitored their receptor-activation activities *in vitro* using HEK293T cells over-expressing NPSR. Because NPS activates NPSR by increasing cAMP production and intracellular Ca^2+^ signaling [9], [10], we used CRE- and NFAT- response elements to measure cAMP and intracellular Ca2+ signaling respectively [35], [36]. As expected, treatment with wide-type NPS led to dose-dependent stimulation of both CRE- and NFAT- luciferase activities (Fig. 2B and C). We also performed the relaxin-LGR7 ligand-receptor pair [37] as a positive control for the CRE-luciferase assay. Also, gastrin-CCKB ligand-receptor pair [38] was used as a positive control for the SRE-luciferase assay. Cells transfected with the empty vector were also treated with NPS to serve as a negative control (Fig. S4).

**Figure 3:**
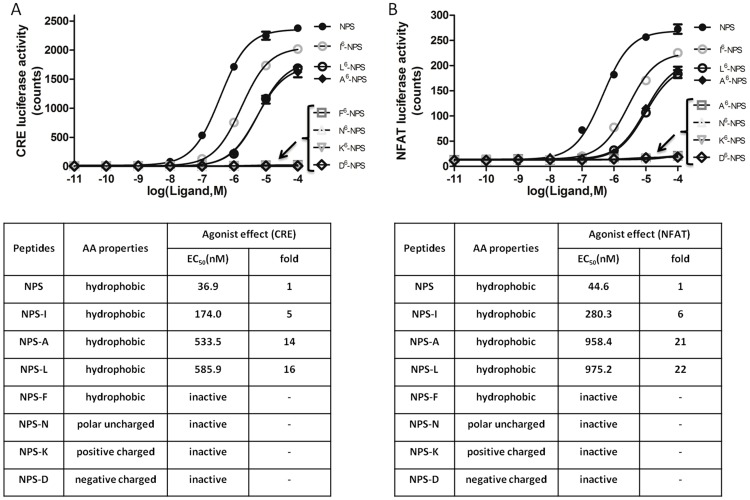
Signaling abilities of different NPS mutants. (**A**) Stimulation of CRE luciferase activity mediated by NPSR in response to increasing dosages of various NPS mutants. (**B**) Stimulation of NFAT luciferase activity mediated by NPSR in response to increasing dosages of various NPS mutants. EC_50_ vales were analyzed using Graphpad Prism 5.0.

As compared with wide-type NPS, the L^6^-NPS variant exhibited lower potencies with apparent EC_50_ values ∼16-fold for CRE-luciferase and ∼22-fold for NFAT-luciferase, respectively (CRE-luciferase activity, apparent EC_50_ values: wild type-37 nM, L^6^-NPS-586 nM; NFAT-luciferase, EC_50_: wild type-45 nM, L^6^-NPS-975 nM) (Fig. 2 and 3). Similar signaling potencies were found when synthetic peptides were obtained from a different source (Fig. S3). Because both valine in the wild type NPS and leucine in the SNP are hydrophobic in nature, these findings suggest that the side chains of 6^th^ residue in NPS are important for NPS signaling transduction.

To further elucidate the importance of the 6^th^ position residue valine for NPS bioactivity, we chemically synthesized additional mutants with substitutions by other amino acids with different chemical properties (alanine-A, isoleucine-I and phenylalanine-F for hydrophobic property; asparagine-N for polar uncharged; lysine-K for positive uncharged; aspartic acid-D for negative charged). Similar to L^6^-NPS, substitution with two other hydrophobic amino acids (A and I) in NPS, showed impaired bioactivity with apparent EC_50_ values ∼5- (I^6^-NPS) and ∼15-fold (A^6^-NPS) higher than the wild type peptide in the CRE-luciferase assay (Fig. 3A). Also, all non-hydrophobic amino acid substitutions (N-, K- and D-) led to a complete loss of bioactivity (Fig. 3A). In contrast to the minimal loss of bioactivity following substitution with the hydrophobic isoleucine-I containing a small side chain, substitution with the hydrophobic phenylalanine-F containing a large side chain showed a complete loss of bioactivity, suggesting a bulky side chain on the 6th position is detrimental for NPS bioactivity. Likewise, similar changes in signaling potencies were found using the NFAT-luciferase assay (Fig. 3B) when peptides with different substitutions were tested. Combining our results with earlier publications [33], [34], it is clear that the 6^th^ position valine plays a critical role in interactions with the NPSR receptor to stimulate downstream signaling pathways.

## Discussion

To minimize the bias in our results, we first used all the publicly available data (the total size of ∼8,000 samples) to calculate the *rs4751440* allele frequency. Secondly, we employed three different methods to investigate the origin of the *rs4751440* allele: a) the estimation of age of polymorphism under neutrality rule; b) the estimation of age of the *rs4751440*-associated haplotypes on the basis of decay of haplotypes; and c) spatially explicit population model and Approximate Bayesian Computation (ABC) inference to estimate the temporal and geographic origin of the *rs4751440* allele. The ABC method not only estimated the age of *rs4751440* allele, but also estimated the location of origin of *rs4751440* allele with addressing population stratification effect [39]–[41]. Also, we performed as many as 3,000,000 simulations to require the best 5,000 and 1,000 simulations to estimate the parameters of origin of *rs4751440* allele, which make the conclusion is more reliable. Based on haplotype analysis and Approximate Bayesian Computation, we concluded that the L^6^-NPS variant probably originated from the ancestor of European population ∼25,000 yrs ago.

NPS, a highly conserved neuropeptide in vertebrates (Fig. 2A), plays important physiological roles in anxiety, fear, hyperactivity, food intake, and sleeping time mediated by its receptor-NPSR [9], [12], [15], [16], [18], [42]. Because the SNP *rs4751440* leads to non-synonymous substitution on the 6^th^ position (V to L) of the NPS mature region and shows ∼13% of allele frequency in Europeans, heterozygous expression of L^6^-NPS is expected in 22% of Europeans whereas 2% of Europeans exclusively express L^6^-NPS (Fig. 1). Thus, 22% of Europeans with heterozygous alleles have decreased NPS bioactivity whereas the 2% of Europeans with homozygous alleles have minimal NPS activity.

Recent murine and human studies suggested the roles of NPS in diverse neural and peripheral functions, including olfaction [43], anxiolytic and anti-depressive effects [44], [45], memory retention [46], monocyte chemotaxis [47], pain-related behaviors [48], neuroendocrine stress responses [49], and panicolytic-like actions [50]. Also, the NPS-NPSR system was found to be involved in addiction-related behaviors including morphine [51] and cocaine addiction [52]–[54]. Furthermore, the NPS-NPSR system interacts with other neural circuitry of the brain. Recent studies showed that the expression pattern of NPS and NPSR is differentially modulated by hyperthyroidism in the rat brain [55]. Also, NPS-NPSR signaling regulates the expression of several other neuropeptides, including cholecystokinin, vasoactive intestinal peptide, peptide YY, and somatostatin [56]. In addition, NPS neurons in the locus coeruleus are activated by stress-related CRF [57]. Following intracerebroventricular injection of NPS, hypothalamic hypocretin-1/orexin-A neurons are activated [58], [59]. These findings suggested that NPS-NPSR signaling affects diverse neural circuitry and brain functions.

Due to the involvement of NPS in various important brain activities, the NPSR antagonists were discovered to antagonize different physiological functions mediated by NPS [53], [60]–[64]. The 20-aa NPS showed large conservation in vertebrate (Fig. 2). Based on structure–activity studies of NPS, the F^2^, R^3^, and N^4^ residues constitute the message domain revealed by the chemical requirements of these positions for NPSR binding and activation. In contrast, the G^5^, V^6^, G^7^ residues are important for shaping the bioactive conformation of the peptide [33], [34]. Further study on G^5^–NPS modification generated the first generation of peptidergic NPSR antagonists, including [d-Cys(tBu)^5^]NPS and [d-Val^5^]NPS whose antagonistic properties were confirmed *in vitro* and *in vivo* [64]–[67]. Also, several non-pepetidergic NPSR antagonists, including SHA 68 [63], RTI-118 [53], QA1 and PI1 [68] have been developed to block various NPS functions. However, some non-peptide antagonists showed less effect *in vivo* than *in vitro* probably due to their poor pharmacokinetic properties [68]. However, the peptidergic analogs appear to be effective both in vitro and in vivo [60], [61], [66]. The V^6^ residue of NPS is located at the NPS hinge region and could be essential for its bioactivity [33], [34]. Our data suggest that the side chain of V6 affects the downstream signaling of NPSR (Fig. 3) and that I^6^-NPS, L^6^-NPS and D^6^-NPS are more potent NPSR agonists. Further modifications could allow the design of agonists and antagonists as therapeutic agents.

With the completion of genome sequencing and the availability of more SNP databases, a number of NPSR gene polymorphism has also been found. Some of the SNPs in the non-coding region were found to affect the NPSR mRNA expression, whereas some SNPs in the NPSR coding region showed reduced NPSR protein membrane trafficking or reduced downstream signaling [69]. In patients, several mutations in the NPSR coding region have been associated with susceptibility to inflammatory bowel diseases [70], asthma pathogenesis [34], obsessive–compulsive disorder [71], fear-potentiated startle [72], modulates response inhibition and error monitoring [73], and macrophage immune responses [74]. It is of interest to investigate possible changes in susceptibility to these diseases in Europeans with hetero-and homozygous SNP *rs4751440* genotypes. Our study also provides the basis for future elucidation of potential phenotypic diversities between European and other populations as related to NPS signaling in the regulation of diverse brain functions.

## Supporting Information

Figure S1 Linkage disequilibrium patterns of genomic regions surrounding *rs4751440*. The region surrounding SNP *rs4751440* was analyzed to include 888 SNPs over a 500 kb span in the CEU (Utah Residents with Northern and Western European ancestry) population based on the HapMap using the Haploview. An arrow denotes the location of SNP *rs4751440*. A ∼14 kb block of linkage disequilibrium covering *rs4751440* is highlighted in green.
(PDF)

Figure S2 Approximate Posterior Density Estimates of Demographic and Evolutionary Parameters, Related to Figure 1B. ABC was performed retaining the top 5,000 simulations among a total of 3,000,000 simulations (tolerance level 0.17%). The posterior density estimates shown in dash blue lines are from the top 1,000 simulations (tolerance level 0.03%).
(PDF)

Figure S3 Lower NPSR receptor signaling ability of the L^6^-NPS variant. Wild type and L^6^-NPS were synthesized by the NEO Group Inc. (**A**) Comparison of NPS and L^6^-NPS signaling based on the CRE-luciferase assay. (**B**) Comparison of NPS and L6-NPS signaling based on the NFAT-luciferase assay. Data were analyzed using Graphpad Prism 5.0.
(PDF)

Figure S4 Positive control and negative control for the CRE- and SRE- luciferase assay. No stimulation on NPS-empty vector pair for the CRE- and SRE- luciferase assay; normal stimulation on relaxin-LGR7 ligand-receptor pair for the CRE- luciferase assay and gastrin-CCKB ligand-receptor pair for the SRE- luciferase assay.
(PDF)

Table S1 SNPs causing non-synonymous substitution in the mature regions of all type A (**Table S1A**) and type B (**Table S1B**) polypeptide ligands found in the Human Plasma Membrane receptome Database.
(XLS)

Table S2 Populations used as observed data point in Figure 1.
(PDF)
