# Supplementary figures and images for: A Single-Nucleotide Polymorphism of Human Neuropeptide S Gene Originated from Europe Shows Decreased Bioactivity
Source: PLoS One. 2013 Dec 27;8(12):e83009. doi: 10.1371/journal.pone.0083009 (PMC3873911; doi:10.1371/journal.pone.0083009)

Fig. S1

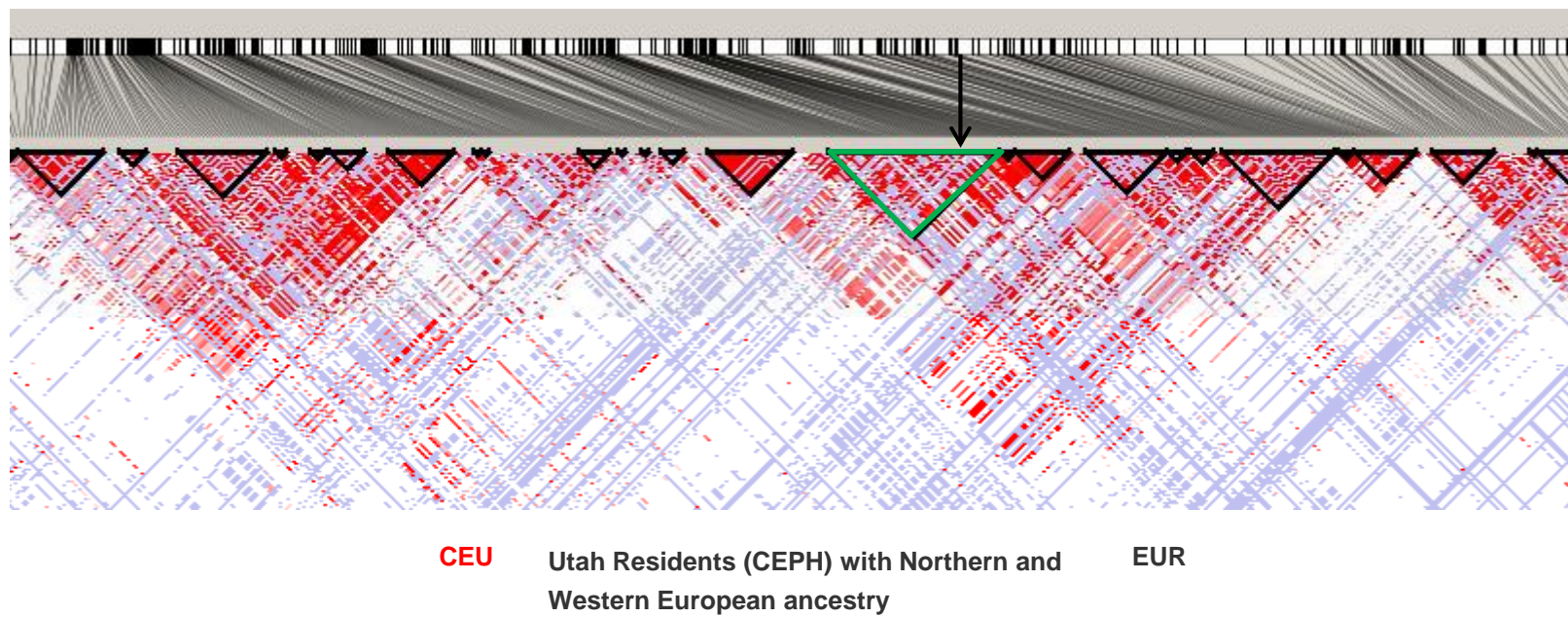

Supplement: Figure S1 — Linkage disequilibrium patterns of genomic regions surrounding rs4751440. The region surrounding SNP rs4751440 was analyzed to include 888 SNPs over a 500 kb span in the CEU (Utah Residents with Northern and Western European ancestry) population based on the HapMap using the Haploview. An arrow denotes the location of SNP rs4751440. A ∼14 kb block of linkage disequilibrium covering rs4751440 is highlighted in green. (PDF) [file pone.0083009.s001.pdf]

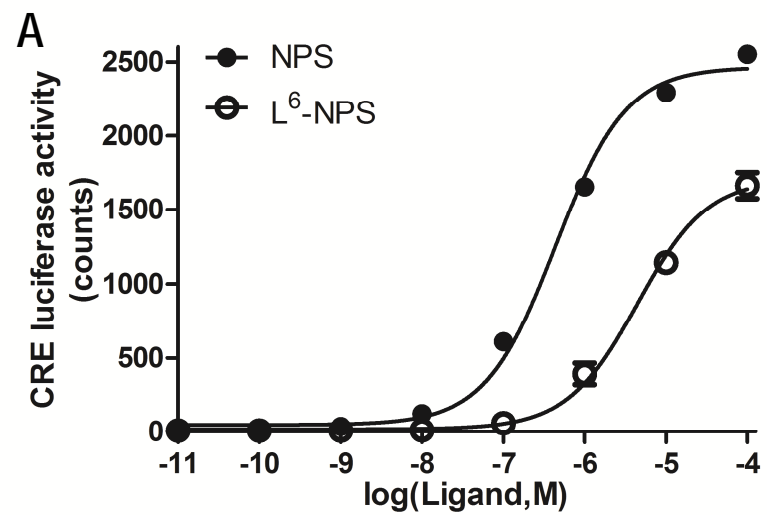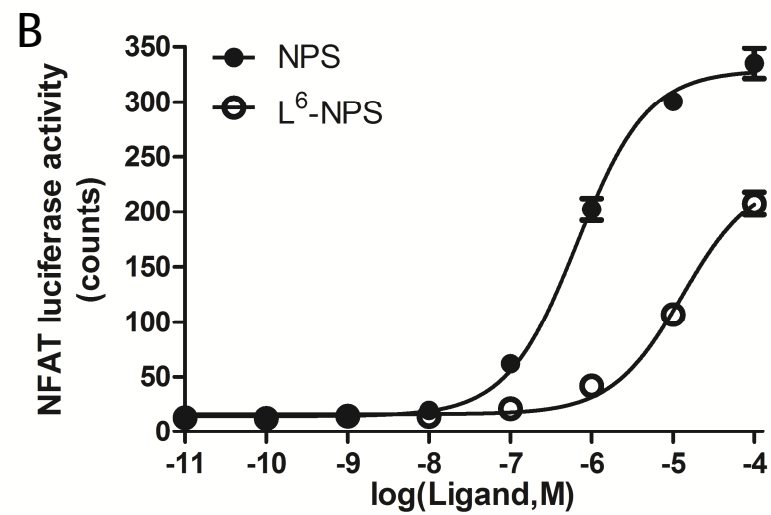

Fig. S3

Supplement: Figure S3 — Lower NPSR receptor signaling ability of the L6-NPS variant. Wild type and L6-NPS were synthesized by the NEO Group Inc. (A) Comparison of NPS and L6-NPS signaling based on the CRE-luciferase assay. (B) Comparison of NPS and L6-NPS signaling based on the NFAT-luciferase assay. Data were analyzed using Graphpad Prism 5.0. (PDF) [file pone.0083009.s003.pdf]

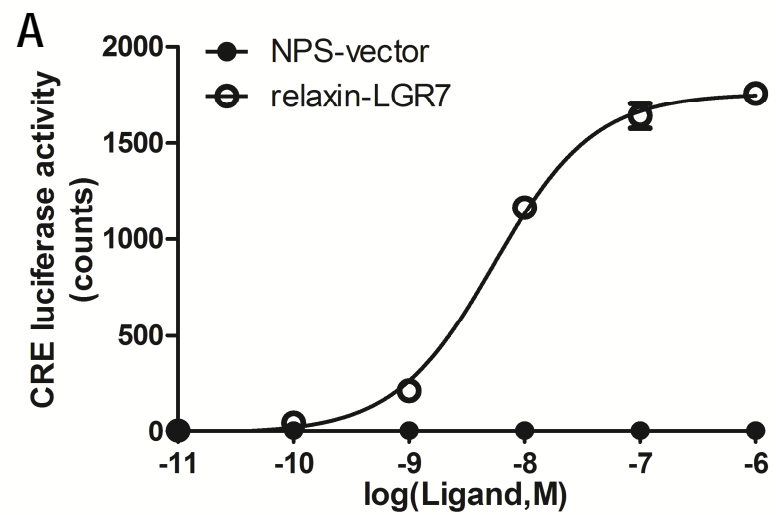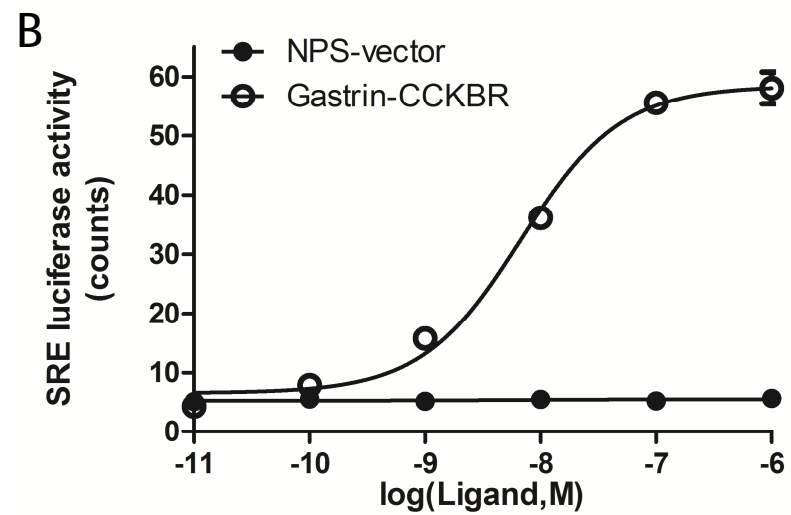

Supplement: Figure S4 — Positive control and negative control for the CRE- and SRE- luciferase assay. No stimulation on NPS-empty vector pair for the CRE- and SRE- luciferase assay; normal stimulation on relaxin-LGR7 ligand-receptor pair for the CRE- luciferase assay and gastrin-CCKB ligand-receptor pair for the SRE- luciferase assay. (PDF) [file pone.0083009.s004.pdf]
