# Supplementary material for: A Single-Nucleotide Polymorphism of Human Neuropeptide S Gene Originated from Europe Shows Decreased Bioactivity
Source: PLoS One. 2013 Dec 27;8(12):e83009. doi: 10.1371/journal.pone.0083009 (PMC3873911; doi:10.1371/journal.pone.0083009)
Supplement: Figure S2 — Approximate Posterior Density Estimates of Demographic and Evolutionary Parameters, Related to Figure 1B. ABC was performed retaining the top 5,000 simulations among a total of 3,000,000 simulations (tolerance level 0.17%). The posterior density estimates shown in dash blue lines are from the top 1,000 simulations (tolerance level 0.03%). (PDF) [file pone.0083009.s002.pdf]

Fig. S2

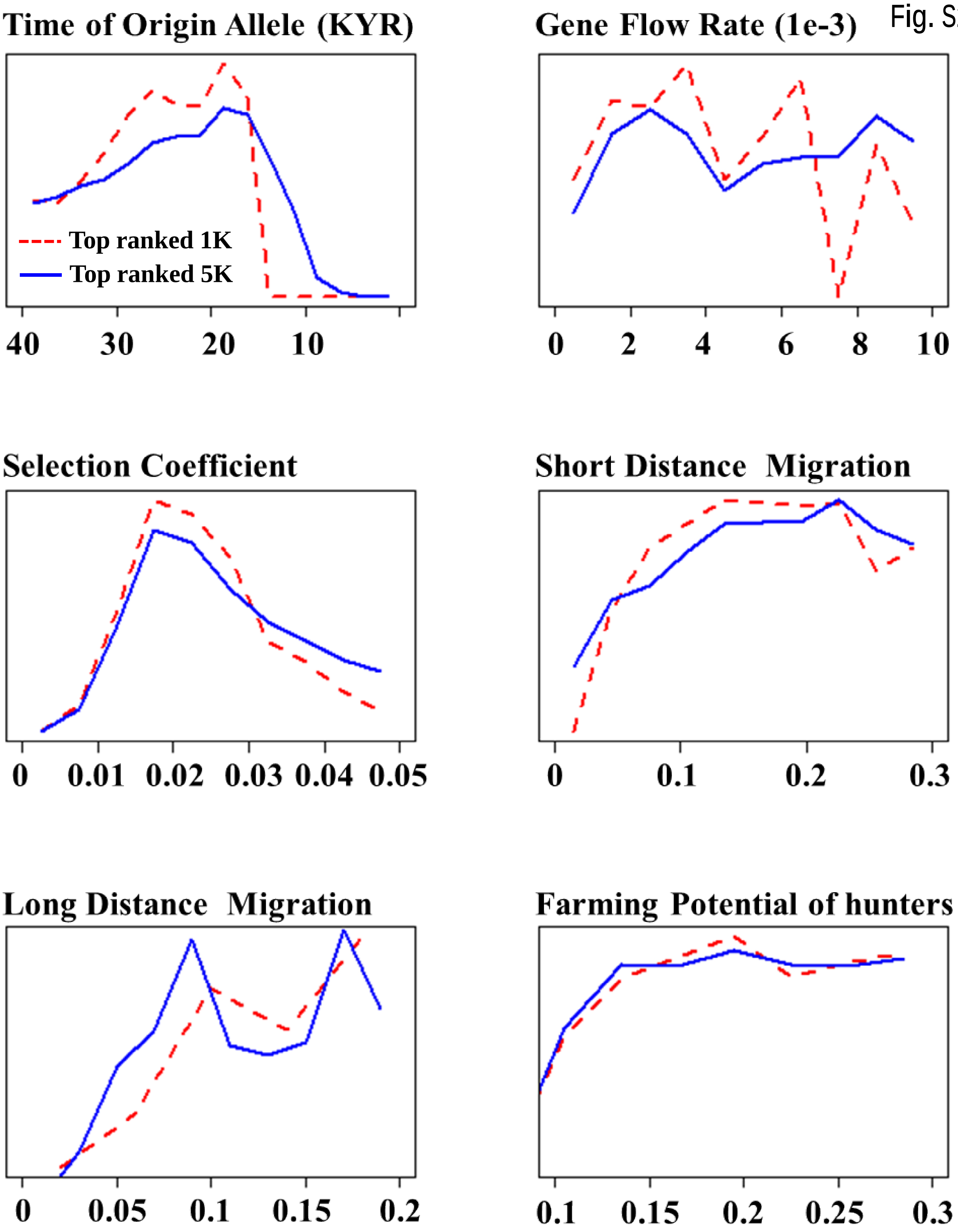
Time of Origin Allele (KYR)
Top ranked 1K
Top ranked 5K
40
30
20
10
Gene Flow Rate (1e-3)
0
2
4
6
8
10
Selection Coefficient
0
0.01
0.02
0.03
0.04
0.05
Short Distance Migration
0
0.1
0.2
0.3
Long Distance Migration
0
0.05
0.1
0.15
0.2
Farming Potential of hunters
0.1
0.15
0.2
0.25
0.3
