# Supplementary material for: A Single-Nucleotide Polymorphism of Human Neuropeptide S Gene Originated from Europe Shows Decreased Bioactivity
Source: PLoS One. 2013 Dec 27;8(12):e83009. doi: 10.1371/journal.pone.0083009 (PMC3873911; doi:10.1371/journal.pone.0083009)
Supplement: Table S2 — Populations used as observed data point in Figure 1. (PDF) [file pone.0083009.s006.pdf]

Table S2. Populations used as observed data point in Figure 1.

Population	Sample Size (2N)	Derived Allele Frequency	Latitude	Longitude
ASW	122	0.016	-28	24
CEU	170	0.147	46	2
CHB	194	0.000	30	112
CHS	200	0.000	21	100
CLM	120	0.050	3	-68
FIN	186	0.070	60	24
GBR	178	0.146	51	0
IBS	28	0.321	40	-3
JPT	178	0.000	38	138
LWK	194	0.000	-3	37
MXL	132	0.076	25	-100
PUR	110	0.036	18	-75
TSI	196	0.112	43	11
YRI	176	0.000	8	5
